# Performance Evaluations on Using Entropy of Ultrasound Log-Compressed Envelope Images for Hepatic Steatosis Assessment: An In Vivo Animal Study

**DOI:** 10.3390/e20020120

**Published:** 2018-02-11

**Authors:** Jui Fang, Ning-Fang Chang, Po-Hsiang Tsui

**Affiliations:** 1 Ph.D. Program in Biomedical Engineering, College of Engineering, Chang Gung University, Taoyuan 33302, Taiwan; 2 Department of Medical Imaging and Radiological Sciences, College of Medicine, Chang Gung University, Taoyuan 33302, Taiwan; 3 Medical Imaging Research Center, Institute for Radiological Research, Chang Gung University and Chang Gung Memorial Hospital at Linkou, Taoyuan 33302, Taiwan; 4 Department of Medical Imaging and Intervention, Chang Gung Memorial Hospital at Linkou, Taoyuan 33305, Taiwan

**Keywords:** ultrasound, hepatic steatosis, Shannon entropy, fatty liver

## Abstract

Ultrasound B-mode imaging based on log-compressed envelope data has been widely applied to examine hepatic steatosis. Modeling raw backscattered signals returned from the liver parenchyma by using statistical distributions can provide additional information to assist in hepatic steatosis diagnosis. Since raw data are not always available in modern ultrasound systems, information entropy, which is a widely known nonmodel-based approach, may allow ultrasound backscattering analysis using B-scan for assessing hepatic steatosis. In this study, we explored the feasibility of using ultrasound entropy imaging constructed using log-compressed backscattered envelopes for assessing hepatic steatosis. Different stages of hepatic steatosis were induced in male Wistar rats fed with a methionine- and choline-deficient diet for 0 (i.e., normal control) and 1, 1.5, and 2 weeks (*n* = 48; 12 rats in each group). In vivo scanning of rat livers was performed using a commercial ultrasound machine (Model 3000, Terason, Burlington, MA, USA) equipped with a 7-MHz linear array transducer (Model 10L5, Terason) for ultrasound B-mode and entropy imaging based on uncompressed (*H*_E_ image) and log-compressed envelopes (*H*_B_ image), which were subsequently compared with histopathological examinations. Receiver operating characteristic (ROC) curve analysis and areas under the ROC curves (AUC) were used to assess diagnostic performance levels. The results showed that ultrasound entropy imaging can be used to assess hepatic steatosis. The AUCs obtained from *H*_E_ imaging for diagnosing different steatosis stages were 0.93 (≥mild), 0.89 (≥moderate), and 0.89 (≥severe), respectively. *H*_B_ imaging produced AUCs ranging from 0.74 (≥mild) to 0.84 (≥severe) as long as a higher number of bins was used to reconstruct the signal histogram for estimating entropy. The results indicated that entropy use enables ultrasound parametric imaging based on log-compressed envelope signals with great potential for diagnosing hepatic steatosis.

## 1. Introduction

Hepatic steatosis is a disease in which excessive fat accumulates to form fatty vacuoles in hepatocytes [1,2,3]. Hepatic steatosis is generally a reversible process [1,2], but it may ultimately progress to become steatohepatitis, fibrosis, and cirrhosis [4,5] if no appropriate control and interventions are applied to diet and lifestyle [5]. Consequently, routine examinations of patients with hepatic steatosis are necessary. Conventionally, liver biopsy is the standard method for determining the stage of hepatic steatosis [6,7]. Liver biopsy is not an ideal method for follow-up because it is invasive and causes discomfort, pain, and complications for patients [6]. Thus, noninvasive imaging methods can be used to provide information associated with hepatic steatosis to assist in diagnosis. Currently, computed tomography [8], magnetic resonance imaging [9], and ultrasound B-mode imaging [10,11,12] are widely used to screen and evaluate hepatic steatosis. Of the aforementioned methods, ultrasound is the first-line imaging modality because of its nonionizing radiation, portability, and real-time capability. However, although ultrasound B-scan allows fast screening and evaluation, image brightness and features are strongly dependent on system parameters and operator experiences, resulting in a subjective diagnosis [10,11,12]. Thus, a relatively objective ultrasound evaluation of hepatic steatosis is required.

The appearance of speckle (granular patterns) in the B-mode images of liver tissues is due to ultrasound scattering, which results from the interaction between acoustic scatterers and the incident wave [13,14]. Based on the randomness of ultrasound scattering, the information of backscattered signals can be described using statistical distribution as a clue for characterizing scatterers in tissues [15,16]. The liver is modeled as a collection of hepatocytes and lobules [17]; thus, hepatic steatosis can be treated as a process in which additional fatty scatterers form in liver tissues, resulting in changes in the microstructure and the corresponding statistical distributions of backscattered signals. Thus, studies have attempted to use statistical models, such as Nakagami distribution [15,18], homodyned-K distribution [19], and acoustic structure quantification [16], for hepatic steatosis assessment. The use of statistical models in ultrasound tissue characterization requires two prerequisites: (i) the ultrasound system must provide raw image data, including either radiofrequency (RF) or envelope signals; and (ii) the raw data must follow the used statistical distribution [20]. This limitation motivated researchers to seek nonmodel-based methods as a more flexible solution for characterizing hepatic steatosis.

In fact, several approaches are available for nonmodel-based estimates (e.g., mean, variance, or texture analysis [21,22]). Among all possibilities, Shannon entropy is a widely-known estimate of signal uncertainty and complexity proposed in information theory [23]. Hughes pioneered entropy use to analyze ultrasound backscattered signals for microstructure quantification [24,25]. The feasibility of using ultrasound entropy to characterize tissues has been explored in some clinical topics, such as monitoring progress in Duchenne muscular dystrophy [26] and detecting cataracts [27]. In particular, ultrasound parametric imaging constructed using entropy was shown to have the ability to visually evaluate hepatic steatosis in humans [28]. The theoretical basis of using entropy imaging in ultrasound liver characterization is derived from the fact that entropy is a function of a variable’s probability density function (PDF) to allow descriptions of the statistical properties of signals. Moreover, estimating entropy does not require the data to follow the specific statistical model, providing an opportunity to realize a more flexible analysis approach without considering the signal distribution.

Before ultrasound entropy imaging can be reliably used in practical applications of hepatic steatosis evaluation, we must address some unanswered questions. In previous studies, entropy estimations were based on raw image data [26,27,28], but not on log-compressed envelope signals used for conventional B-mode imaging. However, ultrasound B-scan remains a mainstream in clinical screening, and most commercial B-scan machines do not provide raw data. Thus, the diagnostic performance of entropy estimated using log-compressed envelopes for assessing hepatic steatosis must be investigated. Second, signal PDF reconstruction is necessary for entropy estimation. To reduce the complexity of signal PDF reconstruction, a statistical histogram can be used as an alternative PDF [27,28]. In this condition, the number of bin (NB) is the key factor for determining the shape of the histogram and the corresponding entropy value. The effect of NB on using entropy to characterize hepatic steatosis is unknown.

This study explored (i) the diagnostic performance of ultrasound entropy constructed using envelope and B-scan (log-compressed envelopes) images for evaluating hepatic steatosis, and (ii) the effect of NB on the entropy imaging of hepatic steatosis. In vivo animal experiments were conducted, and the results showed that entropy imaging based on uncompressed envelopes is effective for detecting variations in hepatic steatosis. Entropy imaging using log-compressed envelopes can facilitate the characterization of hepatic steatosis when NB was set to ≥130.

## 2. Materials and Methods

### 2.1. Animal Preparations

The Institutional Animal Care and Use Committee of Chang Gung University approved the animal study. Forty-eight male Wistar rats weighing 210–250 g (age: six weeks) were used in this study. The rats were housed in standard cages and provided with food and tap water ad libitum. After acclimatization of one week, the rats were divided into four groups (*n* = 12 in each group): one group was fed a normal diet (as normal control) and three groups were fed a methionine- and choline-deficient (MCD) diet (Baker Amino Acid Diet lacking choline and methionine 578 B; TestDiet, Richmond, IN, USA) [29,30,31] for 1, 1.5, and 2 weeks to induce different degrees of hepatic steatosis.

### 2.2. Ultrasound Data Acquisition

A commercial ultrasound scanner (Model 3000, Terason, Burlington, MA, USA) equipped with a 7-MHz linear array transducer (Model 10L5, Terason) was used for in vivo scanning of rat livers. The pulse length of the transducer was approximately 0.7 mm, and the sampling rate of RF signals was 30 MHz. Before measurements, each rat was anesthetized with isoflurane, shaved on the abdomen, and placed in a supine position. Ultrasound examination of rat livers was subsequently performed using transhepatic longitudinal scanning to acquire raw image data, which comprised 256 scan lines of backscattered RF signals returned from the liver parenchyma. The imaging depth was 3 cm, and the focal zone was adjusted to be located at the central part of the liver to reduce the effect of beam divergence. Five independent scans were performed for each liver.

### 2.3. Ultrasound Entropy Imaging

**Figure 1:**
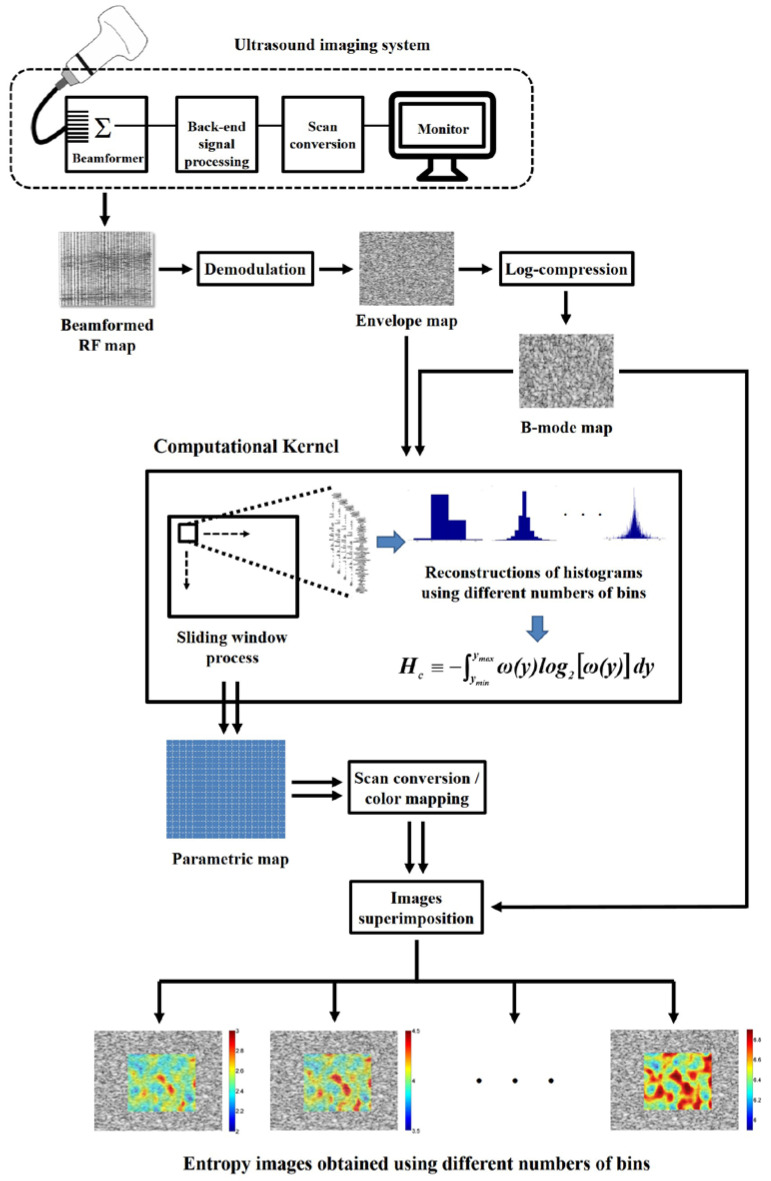
Computational flowchart for ultrasound entropy parametric imaging.

For each raw datum, envelope images were obtained by taking the absolute value of the Hilbert transform of backscattered RF signals [32], and the corresponding B-mode images were formed using logarithm-compressed envelope images with a dynamic range of 40 dB. Subsequently, entropic parametric images were constructed using the sliding window technique to process the envelope and B-mode images. The algorithm of entropy imaging illustrated in Figure 1 comprised the following steps: (i) a square window was placed on the envelope and B-mode images to collect local uncompressed and compressed envelope data. The side length of the window was determined as three times the pulse length (i.e., 2.1 mm), as suggested for ensuring stable estimations of statistical parameters [33]; (ii) the PDFs of local data within the sliding window were obtained using the histograms with different NBs from 10 to 210 in steps of 40 bins for estimating entropy values using Equation (1) [23]:

(1) $$H\equiv -\int_{y_{min}}^{y_{max}}\omega \left(y\right)log_{2}\left[\omega \left(y\right)\right]dy,$$

where *y_min_* and *y_max_* represent the minimal and maximal values of the local data in the window, and *w*(*y*) means the PDF of the data. The entropy values obtained using the envelope (denoted by *H*_E_) and B-mode images (denoted by *H*_B_) were assigned as new pixels located in the center of the window; (iii) the window was moved across the envelope and B-mode images in steps with a 50% window overlap ratio, and Steps (i) to (ii) were repeated to yield *H*_E_ and *H*_B_ parametric images; and (iv) because sliding window processing reduces the image size, interpolations of *H*_E_ and *H*_B_ parametric images were performed. In general, nearest neighbor interpolation allows relatively simple computations with less time consuming. Nevertheless, it may result in distortion of image patterns. A cubic-based interpolation algorithm provides an improved image quality, but the computations are more complex. Compared with the above two methods, linear interpolation can avoid image discontinuity to generate satisfactory results with an acceptable computational efficiency [34]. For this reason, the linear interpolation method was used. Finally, the entropy images were superimposed onto the corresponding B-mode images to provide structural and parametric information.

### 2.4. Histopathological Examinations

After ultrasound scanning, the rats were sacrificed through CO_2_ asphyxiation, and the livers were immediately excised for preparations of formalin-fixed, paraffin-embedded sections stained with hematoxylin-eosin (H&E) and Masson trichrome. Histopathological scores were assigned by an experienced veterinarian blinded to the experimental design according to the scoring systems [35]. Steatosis was graded according to the following scoring system: 0, none (<5% of parenchymal involvement by fat droplets); 1, mild (5–33%); 2, moderate (33–66%); and 3, severe (>66%). Lobular inflammation was graded 0–3 based on the number of inflammatory foci per 200× field (0, no foci; 1, 1–2/200× field; 2, up to 4/200× field; and 3, >4/200× field). Hepatocyte ballooning was graded 0–2 based on an estimate of severity (0, none; 1, few ballooning cells; and 2, prominent ballooning cells). Steatohepatitis was evaluated based on the sum of the following individual grading of three features: steatosis grade (0–3), lobular inflammation grade (0–3), and hepatocellular ballooning grade (0–2). Summed scores of 0–2, 3–4, and >4 indicated no steatohepatitis, borderline cases, and steatohepatitis, respectively. Liver fibrosis was staged using the Metavir scoring system [35] on the basis of the patterns of fibrosis and the increase in connective tissue deposition (F0, no fibrosis; F1, portal fibrosis with no septa; F2, portal fibrosis with few septa; F3, bridging fibrosis with many septa; and F4, cirrhosis).

### 2.5. Statistical Analysis

To avoid interference from large blood vessels, we manually selected a region of interest in the B-mode images to involve corresponding entropy values for averaging. The entropies as a function of steatosis grade were expressed by the box plot, which provides a summary of statistics including median, interquartile range (IQR; a measure of statistical dispersion, being equal to the difference between the 75th and 25th percentiles), range, and data distribution. The diagnostic performances of using *H*_E_ and *H*_B_ parametric images to assess hepatic steatosis were evaluated through receiver operating characteristic (ROC) analysis with 95% confidence intervals. A ROC space is defined by the false positive rate (FPR, or 1-specificity) and the true positive rate (TPR, or sensitivity) as x and y axes, respectively. The TPR describes how many correct positive results (i.e., hepatic steatosis at the stage of interest) occur among all positive samples available in the experiments. The FPR describes how many incorrect positive results occur among all negative samples. The ROC curve was plotted in the ROC space by connecting the points located at (FPR, TPR) obtained using different cutoff values of entropy. The best sensitivity, specificity, and accuracy were also determined using the closest point to (0, 1) on the ROC curve. All statistical analyses were performed using SigmaPlot software (Version 12.0, Systat Software, Inc., San Jose, CA, USA).

## 3. Results

**Figure 2:**
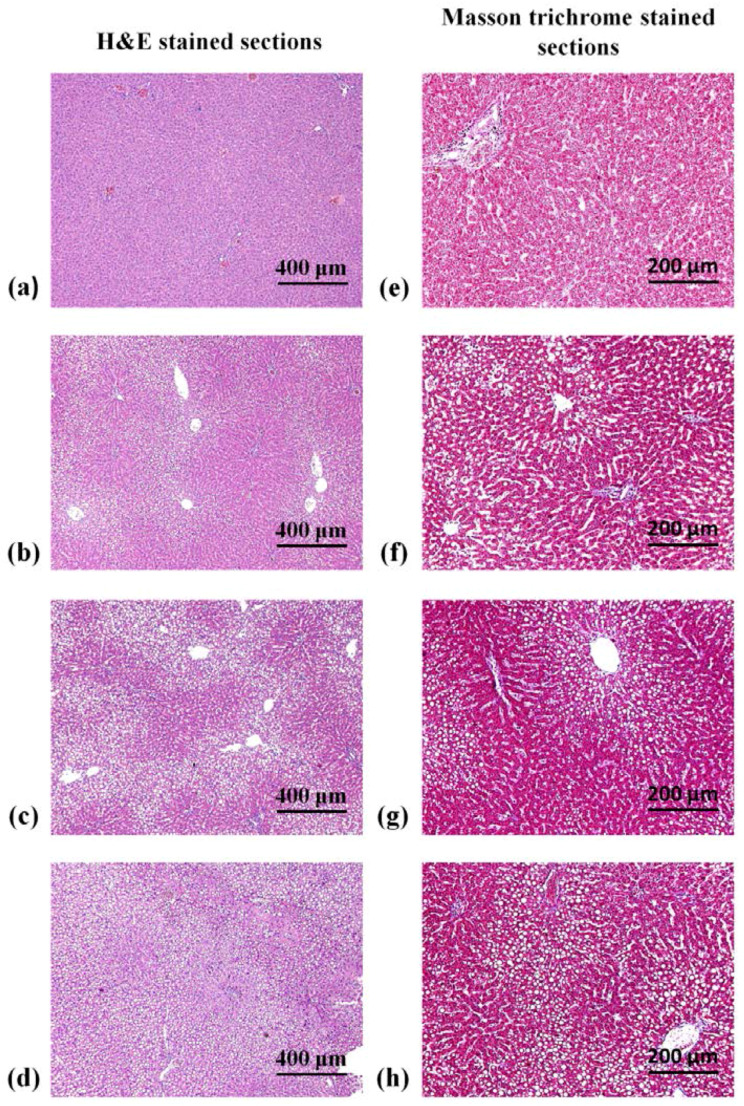
Hematoxylin-eosin- and Masson-stained section images of rat livers for different hepatic steatosis scores. (**a**,**e**) normal; (**b**,**f**) mild; (**c**,**g**) moderate; and (**d**,**h**) severe hepatic steatosis. The concentration of fat droplets gradually increased with hepatic steatosis scores.

Figure 2 shows representative H&E- and Masson-stained images of rat liver sections with different hepatic steatosis scores. The area of fat droplets gradually increased with hepatic steatosis scores, thereby demonstrating the successful induction of hepatic steatosis in rat livers. Table 1 summarizes the changes in the score of each histopathological feature in the normal control and MCD diet-fed groups. Abnormal histopathological features were rarely observed in rat livers in the control group. Hepatic steatosis at scores between 0 and 2 was found after one week of feeding the MCD diet. After 1.5 and 2 weeks of feeding the MCD diet, advanced hepatic steatosis at scores between 1 and 3 were achieved. Concurrently, steatohepatitis scores increased with the duration of feeding the MCD diet; however, they still belonged to mild steatohepatitis. No fibrosis was observed in all the groups.

**Figure 3:**
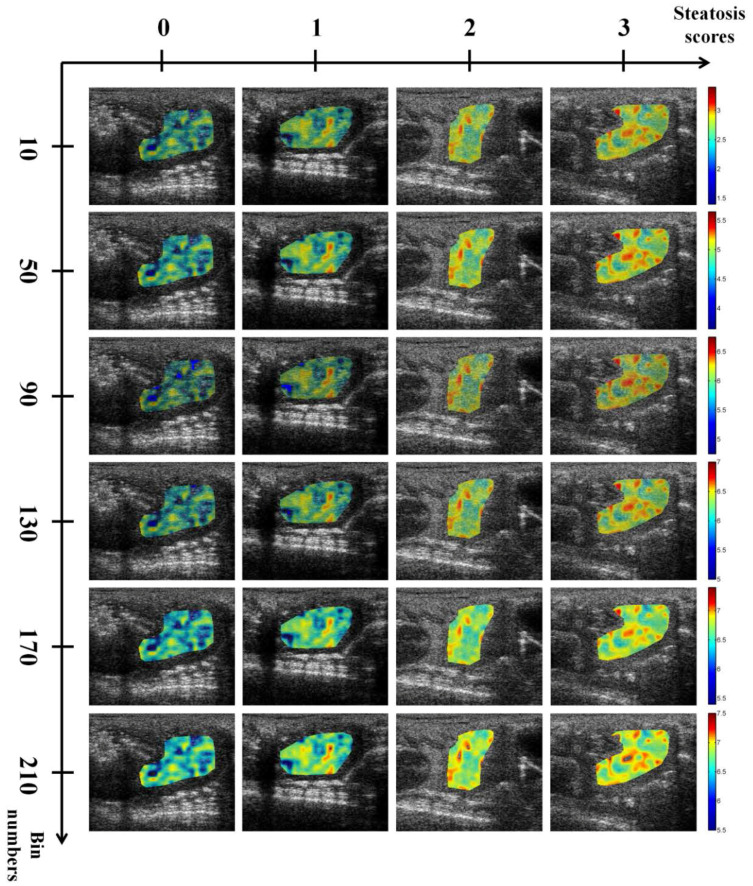
Entropy images obtained using uncompressed envelope images of rat livers with different hepatic steatosis scores and NB. The brightness of the entropy images increased with hepatic steatosis scores. The size of each image is 30 mm (depth) × 38.3 mm (width).

**Figure 4:**
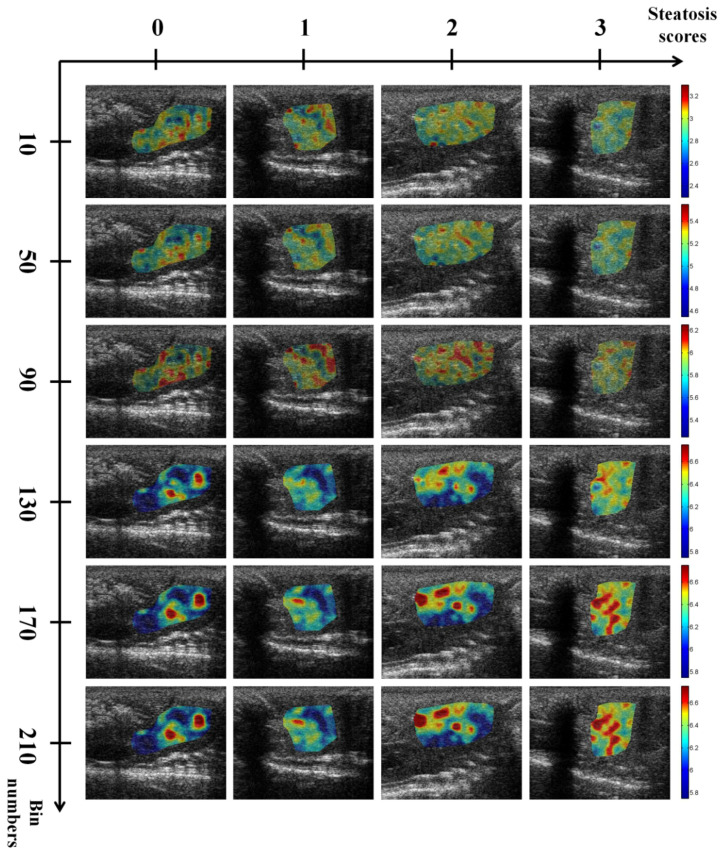
Entropy images obtained using log-compressed envelope images of rat livers with different hepatic steatosis scores and NB. The brightness of the entropy images increased with the hepatic steatosis score when NB ≥ 130. The size of each image is 30 mm (depth) × 38.3 mm (width).

**Figure 5:**
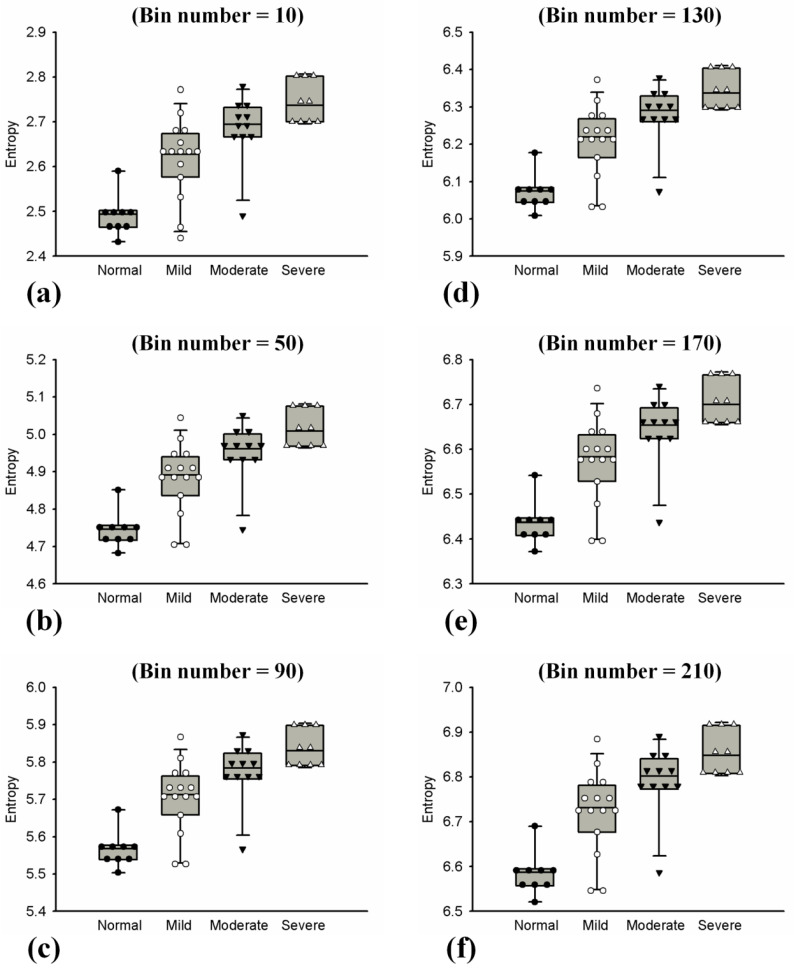
Median and IQR of entropy obtained using uncompressed envelope images with different hepatic steatosis scores and NB. The entropy values increased with hepatic steatosis score regardless of the settings of NB. (**a**) NB = 10; (**b**) NB = 50; (**c**) NB = 90; (**d**) NB = 130; (**e**) NB = 170; (**f**) NB = 210.

**Figure 6:**
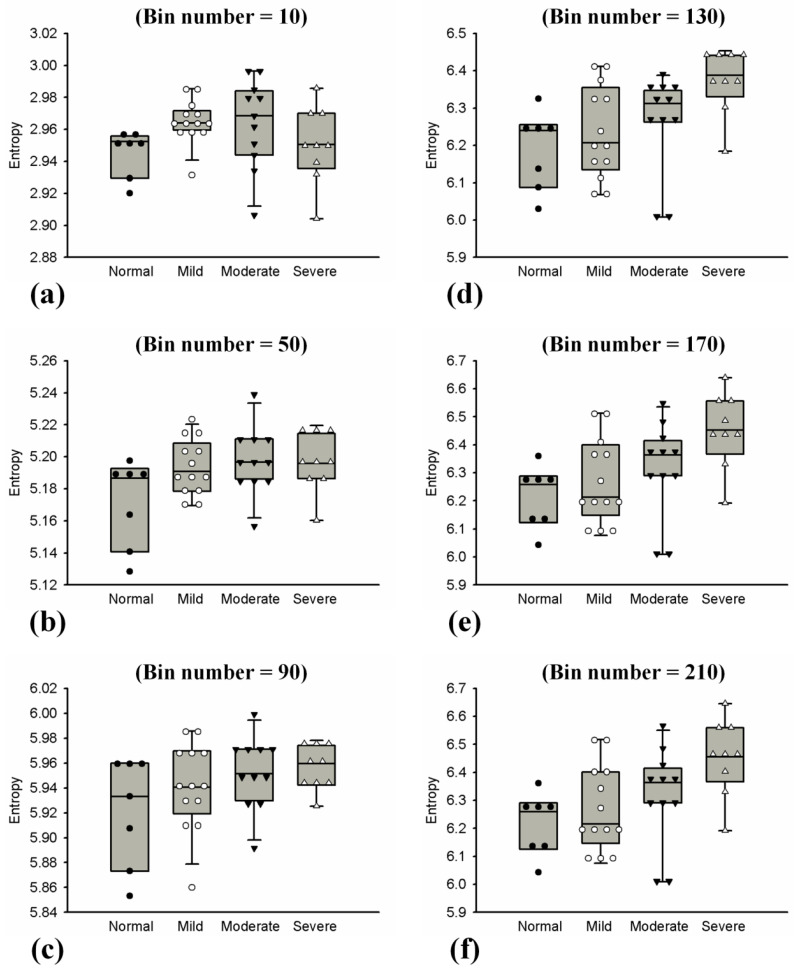
Median and IQR of entropy obtained using log-compressed envelope images with different hepatic steatosis scores and NB. The values increased with hepatic steatosis scores when NB ≥ 130. (**a**) NB = 10; (**b**) NB = 50; (**c**) NB = 90; (**d**) NB = 130; (**e**) NB = 170; (**f**) NB = 210.

**Figure 7:**
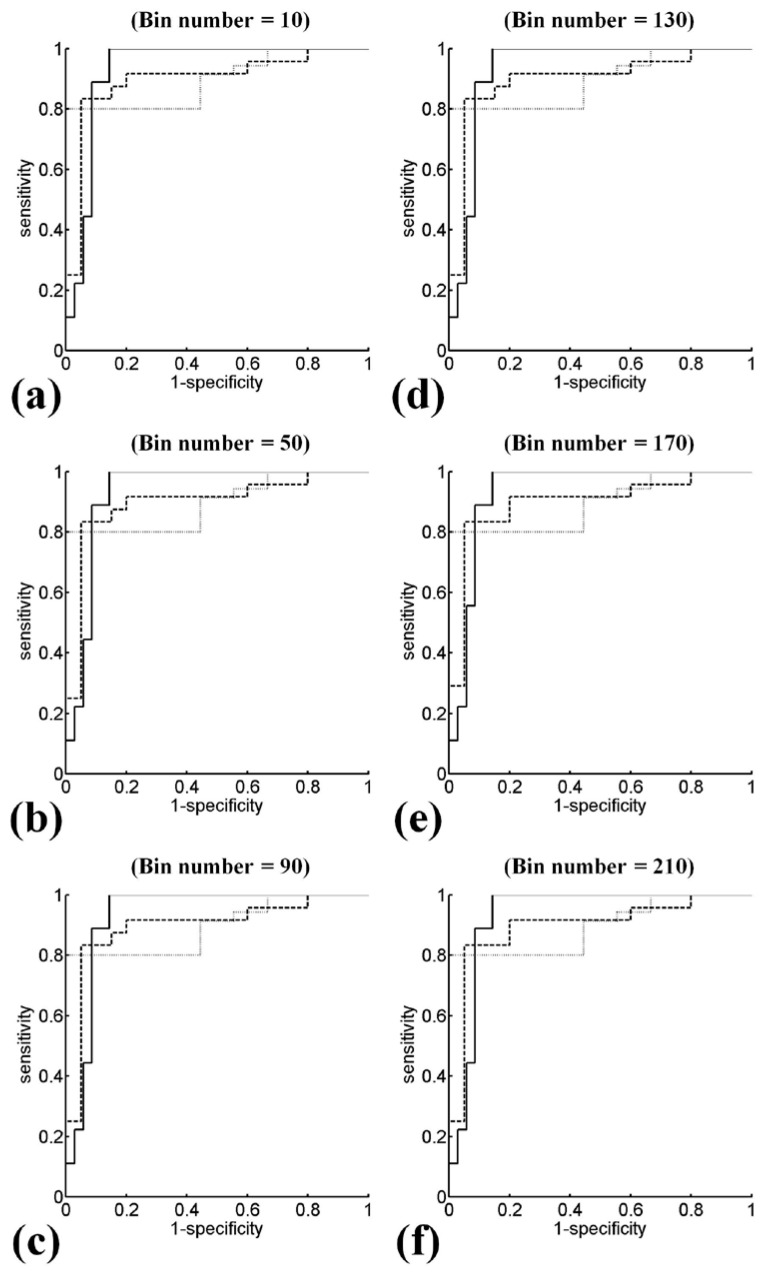
ROC curves of entropy imaging obtained using uncompressed envelopes for diagnosing different steatosis scores. Using NBs from 10 to 210 in steps of 40 bins, the AUCs of (≥mild, ≥moderate, ≥severe) were (0.9302, 0.8987, 0.8952), (0.9316, 0.8979, 0.8952), (0.9302, 0.8979, 0.8952), (0.9302, 0.8979, 0.8952), (0.9302, 0.8979, 0.8952), and (0.9302, 0.8979, 0.8952), respectively. Diagnostic performance is less affected by the settings of NB. (**a**) NB = 10; (**b**) NB = 50; (**c**) NB = 90; (**d**) NB = 130; (**e**) NB = 170; (**f**) NB = 210.

**Figure 8:**
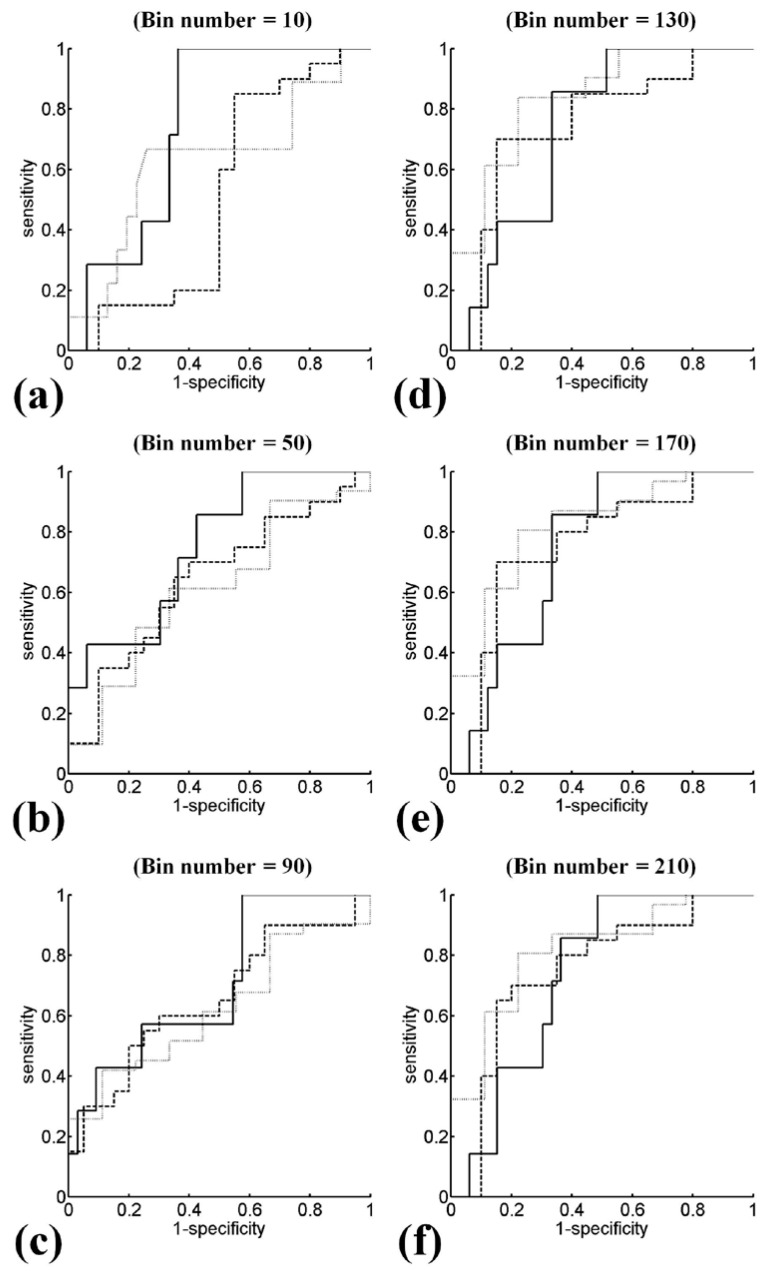
ROC curves of entropy imaging obtained using log-compressed envelope images for diagnosing different steatosis scores. Using NBs from 10 to 210 in steps of 40 bins, the AUCs of (≥mild, ≥moderate, ≥severe) were (0.7489, 0.51, 0.629), (0.7352, 0.6425, 0.6129), (0.7056, 0.6575, 0.6237), (0.7359, 0.7425, 0.8351), (0.7446, 0.75, 0.8172), and (0.7593, 0.7475, 0.8136), respectively. The improved diagnostic performances can be obtained when NB ≥ 130. (**a**) NB = 10; (**b**) NB = 50; (**c**) NB = 90; (**d**) NB = 130; (**e**) NB = 170; (**f**) NB = 210.

Ultrasound *H*_E_ and *H*_B_ images with scores of hepatic steatosis ranging from 0 to 3 constructed using different NBs are shown in Figure 3 and Figure 4, respectively. Using different NBs, the brightness of the *H*_E_ images increased with the scores of hepatic steatosis, but the *H*_B_ image could not show changes in the degree of hepatic steatosis when NB was <130. Figure 5 and Figure 6 show the quantitative analysis of the medians and IQRs of *H*_E_ and *H*_B_ as a function of steatosis score obtained using different NBs. Although NB affects the estimations of *H*_E_ and *H*_B_ values, the *H*_E_ value always monotonically increased with the stage of hepatic steatosis, regardless of the NB. However, no significant relationship between *H*_B_ value and the stage of steatosis was found when NB was <130, implying that the diagnostic performance of the *H*_B_ image is sensitive to NB, as supported by the results in Figure 7 and Figure 8. Using NB = 10, the AUCs of (*H*_E_, *H*_B_) were (0.93, 0.75), (0.89, 0.51), and (0.89, 0.63) for diagnosing the steatosis stages of mild, moderate, and severe, respectively. The AUCs of (*H*_E_, *H*_B_) were (0.93, 0.74), (0.89, 0.74), and (0.89, 0.84) for diagnosing the steatosis stages of mild, moderate, and severe, respectively, when NB was 130. Table 2 and Table 3 summarize the diagnostic performance of the *H*_B_ and *H*_E_ images. The results suggested that ultrasound entropy imaging using *H*_E_ performed effectively in hepatic steatosis assessment. The diagnostic performance of the *H*_B_ image remained stable if NB was ≥130.

## 4. Discussion

### 4.1. Significance of This Study

Entropy is a nonmodel-based approach that allows the analysis of signal uncertainty and complexity. In the past, entropy was not applied to ultrasound parametric imaging using log-compressed backscattered signals, which are typically used for general B-mode imaging of livers. In this study, we performed animal experiments in vivo to explore the feasibility of using ultrasound entropy imaging based on uncompressed and compressed envelopes for quantifying hepatic steatosis. As revealed in the Results section, *H*_E_ imaging is less affected by the effect of NB for detecting variations in the stage of hepatic steatosis. More importantly, *H*_B_ imaging also performed effectively in hepatic steatosis assessment as long as a higher NB was used to construct the signal PDF. This is the first study to demonstrate that information entropy enables ultrasound parametric imaging based on log-compressed envelope data with the ability to characterize hepatic steatosis.

### 4.2. Effects of Hepatic Steatosis On Entropy

The sources of ultrasound scattering in the liver tissue are both diffuse and coherent. Hepatocytes (side lengths of 20–30 μm) and many small vessels within the liver are only several tenths of a millimeter in diameter and are diffuse scattering objects [36,37]. The portal triads (each of which contains a bile duct, a portal venule, a portal arteriole, and lymphatic vessels) are considered a source of coherent ultrasound scattering in the liver [17]. The liver lobule is a polygonal mass with three to six portal triads and comprises a central vein surrounded by plates and hepatocytes. Therefore, the liver lobule may be considered the most active scattering unit, comprising both simultaneous diffused and coherent scatterers, when interpreting interactions between ultrasound waves and the liver tissue [17]. During hepatic steatosis formation, the vacuole of fat fills the hepatocytes and displaces the nuclei to the periphery; thus, hepatic steatosis is similar to a process of increasing the scatterer number within the liver parenchyma [15,28]. Under this condition, various echo amplitudes exist and signal uncertainty and unpredictability (entropy) increase. This is why the results showed that the entropy values (*H*_E_ and *H*_B_) are proportional to the hepatic steatosis score.

### 4.3. Effects of NB on Entropy

The current results indicated that the estimated entropy value depends on the setting of the NB. The diagnostic performances of *H*_E_ and *H*_B_ imaging are also different. As shown in Table 2 and Table 3, the AUCs of *H*_E_ imaging in identifying different stages of hepatic steatosis are almost independent of NB (at least in the range we tested in this study) and remained the same when NB ≥ 90. However, the setting of NB strongly affects *H*_B_ imaging to characterize hepatic steatosis. Before explaining how NB affects the performance of entropy imaging based on log-compressed envelopes, we should first discuss the role of NB in signal PDF reconstruction. In principle, a histogram is an accurate representation of the distribution of numerical data and is an estimate of the probability distribution of a continuous variable. To ensure that the shape of a histogram approximates that of the signal PDF, appropriate settings of NB are necessary to avoid distorting the reconstructed histogram. Essentially, NB plays the role of the sampling rate for the probability distribution of a signal. According to the sampling theory (i.e., Nyquist theory), twice the signal frequency is the minimum sampling rate required for capturing all the information from a signal [38,39]. The concept of sampling theory provides us with more profound understanding that using a higher NB facilitates reconstructing the signal histogram to improve entropy imaging performance in tissue characterization, although determining the cutoff NB that maximizes entropy diagnostic performance is currently difficult. Moreover, according to observations of the experimental results, the minimum requirement of NB seemingly also depends on the dynamic range of envelope signals. Compared with the compressed envelope data, the uncompressed envelopes have a larger dynamic range in amplitude, which is favorable for using a lower NB to satisfy a correct description of the signal amplitude distribution. In this condition, increasing the NB only influences the values of estimated entropies, but does not contribute any improvements on diagnostic performances. Contrarily, the compressed envelopes have a smaller dynamic range in amplitude due to the log transformation processing of the backscattered data for enhancing the contrast of weak echoes [40,41]. As expected, the resolution of the histogram constructed using a low NB may be insufficiently high to describe the PDF of the signal amplitude. This is why the diagnostic performance of ultrasound entropy imaging based on *H*_B_ in evaluating hepatic steatosis is more sensitive to the NB.

While increasing the NB for improving the performance of ultrasound entropy imaging, we should also note that the NB cannot be set too large. The data in Table 3 showed that accuracies slightly decreased when using NB > 130. In the present work, very large bin numbers were not used; however, continuously increasing the NB to infinity will make the signal statistics approximate the uniform distribution [42], which is unworkable in tissue characterization. In other words, besides the minimum requirement of NB, the upper limit for the NB may also be a critical consideration.

### 4.4. Future Challenges and Work

Typically, real-time access of ultrasound RF data in the hardware is difficult because it needs sufficient memory and a transfer rate between the acquisition board and the workstation. However, the data access of log-compressed envelopes is relatively simple because techniques of down-sampling have been frequently used in modern system design. The current study enables visualizing changes in the microstructures of the liver parenchyma with steatosis using entropy of log-compressed backscattered data. Thus, ultrasound entropy imaging may be more suitable to be combined with general B-scan machines for routine examinations and follow-up of patients with hepatic steatosis. Prior to applying entropy imaging to clinical examinations, some challenges must be overcome in the future. Different systems may use different techniques to perform log-compression (e.g., using a look-up table or real-time computation on a chip). Some specifications (e.g., the bit number of the analog-to-digital converter for data acquisition) and parameters for postprocessing (e.g., scan conversion and gray-level adjustment) also affect the dynamic range of signals. Further investigations of ultrasound entropy imaging using different data formats are necessary.

## 5. Conclusions

In this study, we applied Shannon entropy to ultrasound parametric imaging based on log-compressed backscattered signals as a new solution for assessing hepatic steatosis. The effects of NB on the diagnostic performances of entropy imaging in scoring the stage of hepatic steatosis were also explored. The results obtained from the in vivo model highlighted two key findings: (i) ultrasound entropy imaging constructed using uncompressed backscattered envelopes (*H*_E_ image) is less affected by the NB, providing a promising ability to differentiate different stages of hepatic steatosis, and (ii) entropy use allows ultrasound parametric imaging based on log-compressed envelope data, which performs effectively in characterizing hepatic steatosis as long as a high NB is used to reconstruct the signal histogram for estimating entropy. This study facilitates the statistical analysis of ultrasound backscattering for hepatic fat characterization using log-compressed backscattered data. Future work on ultrasound entropy imaging using different formats of compressed data is suggested before clinical applications in practice.

## Figures and Tables

**Table 1:** Changes in steatosis score, steatohepatitis score, and fibrosis stage relative to the duration of MCD diet.

	Control Group	MCD for 1 Week	MCD for 1.5 Weeks	MCD for 2 Weeks
Steatosis (score, 0–3)	0, 0, 0, 1, 1, 1, 0, 0, 0, 0, 0, 0	1, 1, 1, 0, 2, 1, 1, 1, 1, 2, 1, 1	3, 1, 3, 2, 2, 1, 3, 2, 2, 2, 2, 2	3, 3, 1, 2, 3, 1, 3, 3, 2, 2, 3, 2
Lobular inflammation (score, 0–3)	0, 0, 0, 0, 1, 1, 0, 0, 0, 0, 0, 0	1, 1, 1, 1, 1, 0, 1, 0, 1, 0, 0, 0	1, 1, 1, 0, 1, 1, 1, 1, 0, 0, 0, 0	1, 0, 1, 0, 1, 0, 1, 1, 0, 0, 0, 0
Hepatocyte ballooning (score, 0–2)	0, 0, 0, 0, 0, 0, 0, 0, 0, 0, 0, 1	1, 1, 0, 0, 1, 1, 1, 0, 1, 2, 1, 0	1, 1, 1, 1, 0, 1, 0, 1, 1, 1, 0, 0	1, 1, 1, 1, 1, 1, 1, 1, 1, 1, 0, 1
Steatohepatitis (score, 0–8)	0, 0, 0, 1, 2, 2, 0, 0, 0, 0, 0, 1	3, 3, 2, 1, 4, 2, 3, 1, 3, 4, 2, 1	5, 3, 5, 3, 3, 3, 4, 4, 3, 3, 2, 2	5, 4, 3, 3, 5, 2, 5, 5, 3, 3, 3, 3
Fibrosis (stage, 0–4)	0, 0, 0, 0, 0, 0, 0, 0, 0, 0, 0, 0	0, 0, 0, 0, 0, 0, 0, 0, 0, 0, 0, 0	0, 0, 0, 0, 0, 0, 0, 0, 0, 0, 0, 0	0, 0, 0, 0, 0, 0, 0, 0, 0, 0, 0, 0

Note: steatohepatitis = the unweighted sum of the score of steatosis (score, 0–3), lobular inflammation (score, 0–3), and hepatocyte ballooning (score, 0–2) and thus ranges from 0 to 8.

**Table 2:** Performance levels of entropy in the diagnosis of hepatic steatosis (obtained using uncompressed envelope images).

	Cut off Value	AUC (95% CI)	Sensitivity (%)	Specificity (%)	PPV (%)	NPV (%)	Accuracy (%)
Bin number = 10							
≥mild	2.5093	0.9302 (0.8106–1)	88.8889	91.4286	72.7273	96.9697	90.9091
≥moderate	2.6468	0.8979 (0.8029–1)	83.3333	95	95.2381	82.6087	88.6364
≥severe	2.6945	0.8952 (0.7984–0.992)	80	100	100	56.25	84.0909
Bin number = 50							
≥mild	4.7636	0.9316 (0.8106–1)	88.8889	91.4286	72.7273	96.9697	90.9091
≥moderate	4.9913	0.8979 (0.8029–0.9929)	83.3333	95	95.2381	82.6087	88.6364
≥severe	4.9616	0.8952 (0.7984–0.992)	80	100	100	56.25	84.0909
Bin number = 90							
≥mild	5.5846	0.9302 (0.8106–1)	88.8889	91.4286	72.7273	96.9697	90.9091
≥moderate	5.7329	0.8979 (0.8029–0.9929)	83.3333	95	95.2381	82.6087	88.6364
≥severe	5.784	0.8952 (0.7984–0.992)	80	100	100	56.25	84.0909
Bin number = 130							
≥mild	6.0903	0.9302 (0.8106-1)	88.8889	91.4286	72.7273	96.9697	90.9091
≥moderate	6.2392	0.8979 (0.8029–0.9929)	83.3333	95	95.2381	82.6087	88.6364
≥severe	6.2901	0.8952 (0.7984–0.992)	80	100	100	56.25	84.0909
Bin number = 170							
≥mild	6.4544	0.9302 (0.8106–1)	88.8889	91.4286	72.7273	96.9697	90.9091
≥moderate	6.6021	0.8979 (0.8029–0.9929)	83.3333	95	95.2381	82.6087	88.6364
≥severe	6.6535	0.8952 (0.7984-0.992)	80	100	100	56.25	84.0909
Bin number = 210							
≥mild	6.6027	0.9302 (0.8106-1)	88.8889	91.4286	72.7273	96.9697	90.9091
≥moderate	6.7518	0.8979 (0.8029-0.9929)	83.3333	95	95.2381	82.6087	88.6364
≥severe	6.8022	0.8952 (0.7984-0.992)	80	100	100	56.25	84.0909

PPV: positive predictive value; NPV: negative predictive value, AUC: area under the receiver operating characteristics curve.

**Table 3:** Performance levels of entropy in the diagnosis of hepatic steatosis (obtained using log-compressed envelope images).

	Cut off Value	AUC (95% CI)	Sensitivity (%)	Specificity (%)	PPV (%)	NPV (%)	Accuracy (%)
Bin number = 10							
≥mild	2.9578	0.7489 (0.5231–0.9747)	100	63.6364	36.8421	100	70
≥moderate	2.9683	0.51 (0.3246–0.6954)	85	45	60.7143	75	65
≥severe	2.9511	0.629 (0.4074–0.8507)	66.6667	74.1935	42.8571	88.4615	72.5
Bin number = 50							
≥mild	5.1926	0.7532 (0.5286–0.9979)	85.7143	57.5758	30	95	62.5
≥moderate	5.1926	0.6425 (0.467–0.818)	65	65	65	65	65
≥severe	5.1936	0.6129 (0.4071–0.8187)	61.2903	66.6667	86.3636	33.3333	62.5
Bin number = 90							
≥mild	5.9331	0.7056 (0.4701–0.9411)	57.1429	75.7576	33.3333	89.2857	72.5
≥moderate	5.9463	0.6575 (0.4841–0.8308)	60	70	66.6667	63.3636	65
≥severe	5.9467	0.6237 (0.4201-0.8272)	51.6129	66.6667	84.2105	28.5714	55
Bin number = 130							
≥mild	6.2553	0.7359 (0.5069–0.965)	85.7143	66.6667	35.2941	95.6521	70
≥moderate	6.2253	0.7425 (0.5857–0.8993)	70	85	82.3529	73.913	77.5
≥severe	6.3475	0.8351 (0.7033–0.967)	83.871	77.7778	92.8571	58.3333	82.5
Bin number = 170							
≥mild	6.2879	0.7446 (0.5176–0.9715)	85.7143	66.6667	35.2941	95.6522	70
≥moderate	6.2879	0.75 (0.595–0.905)	70	85	82.3529	73.913	77.5
≥severe	6.3914	0.8172 (0.6771–0.9573)	80.6451	77.7778	92.5926	53.8461	80
Bin number = 210							
≥mild	6.2907	0.7593 (0.5069–0.965)	85.7143	63.6364	33.3333	95.4545	67.5
≥moderate	6.2907	0.7475 (0.5919–0.9031)	70	80	77.7778	72.7273	75
≥severe	6.3946	0.8136 (0.6719-0.9553)	80.6452	77.7778	92.5926	53.8462	80

PPV: positive predictive value; NPV: negative predictive value, AUC: area under the receiver operating characteristics curve.
